# High HbA1c levels correlate with reduced plaque regression during statin treatment in patients with stable coronary artery disease: Results of the coronary atherosclerosis study measuring effects of rosuvastatin using intravascular ultrasound in Japanese subjects (COSMOS)

**DOI:** 10.1186/1475-2840-11-87

**Published:** 2012-07-25

**Authors:** Hiroyuki Daida, Tadateru Takayama, Takafumi Hiro, Masakazu Yamagishi, Atsushi Hirayama, Satoshi Saito, Tetsu Yamaguchi, Masunori Matsuzaki

**Affiliations:** 1 Department of Cardiology, Juntendo University School of Medicine, 2-1-1 Hongo, Bunkyo-ku, Tokyo, 113-8421, Japan; 2 Division of Cardiology, Nihon University School of Medicine, Tokyo, Japan; 3 Kanazawa University Graduate School of Medicine, Kanazawa, Japan; 4 Division of Cardiology, Keiai Hospital, Tokyo, Japan; 5 Toranomon Hospital, Tokyo, Japan; 6 Division of Cardiology, Department of Medicine and Clinical Science, Yamaguchi University Graduate School of Medicine, Ube, Japan

**Keywords:** Atherosclerosis, Coronary artery disease, Intravascular ultrasound, HbA1c, Rosuvastatin

## Abstract

**Background:**

The incidence of cardiac events is higher in patients with diabetes than in people without diabetes. The Coronary Atherosclerosis Study Measuring Effects of Rosuvastatin Using Intravascular Ultrasound in Japanese Subjects (COSMOS) demonstrated significant plaque regression in Japanese patients with chronic coronary disease after 76 weeks of rosuvastatin (2.5 mg once daily, up-titrated to a maximum of 20 mg/day to achieve LDL cholesterol <80 mg/dl).

**Methods:**

In this subanalysis of COSMOS, we examined the association between HbA1c and plaque regression in 40 patients with HbA1c ≥6.5% (high group) and 86 patients with HbA1c <6.5% (low group).

**Results:**

In multivariate analyses, HbA1c and plaque volume at baseline were major determinants of plaque regression. LDL cholesterol decreased by 37% and 39% in the high and low groups, respectively, while HDL cholesterol increased by 16% and 22%, respectively. The reduction in plaque volume was significantly (p = 0.04) greater in the low group (from 71.0 ± 39.9 to 64.7 ± 34.7 mm^3^) than in the high group (from 74.3 ± 34.2 to 71.4 ± 32.3 mm^3^). Vessel volume increased in the high group but not in the low group (change from baseline: +4.2% vs −0.8%, p = 0.02). Change in plaque volume was significantly correlated with baseline HbA1c.

**Conclusions:**

Despite similar improvements in lipid levels, plaque regression was less pronounced in patients with high HbA1c levels compared with those with low levels. Tight glucose control during statin therapy may enhance plaque regression in patients with stable coronary disease.

**Trial registration:**

ClinicalTrials.gov, Identifier NCT00329160

## Background

Intravascular ultrasound (IVUS) has been widely applied in recent clinical trials focusing on coronary atherosclerosis [1-6] and has provided new insights into the pathophysiology of atherosclerotic plaque progression and regression.

Plaque regression has been documented in several studies using IVUS to investigate the effects of lipid-lowering therapies, as well as those of anti-hypertensive drugs and anti-diabetic drugs [7-10]. The results of those studies indicate that plaque regression is influenced by several clinical factors, including lipid profiles, diabetic status and blood pressure. Interestingly, diabetic status appears to be one of the major determinants of plaque progression and/or regression [11]. Moreover, diabetes mellitus is an important residual risk factor for prevention of atherosclerotic disease following LDL cholesterol (LDL-C)-lowering therapy [12].

HbA1c has long been used as a marker for glycaemic control in patients with diabetes mellitus and in people with prediabetes [13]. Several epidemiological studies have shown a positive association between HbA1c and risk of cardiovascular disease [14,15]. However, it is unknown whether glucose control is associated with the change in plaque volume following interventions to treat dyslipidaemia, for example.

The Coronary Atherosclerosis Study Measuring Effects of Rosuvastatin Using Intravascular Ultrasound in Japanese Subjects (COSMOS, ClinicalTrials.gov Identifier: NCT 00329160) was a multicentre, open-label trial conducted in 37 institutions in Japan. The study examined the effects of rosuvastatin on plaque volume in 214 hypercholesterolaemic patients with stable coronary artery disease using IVUS [5,6]. In this trial, rosuvastatin decreased LDL-C and increased HDL cholesterol (HDL-C), which significantly reduced plaque volume by 5%. Considering the limited understanding of whether glucose control is associated with changes in plaque volume, we conducted a subanalysis of the COSMOS study to analyse the relationship between HbA1c and change in plaque volume following 76 weeks of treatment with rosuvastatin.

## Methods

### Study design

The study design and primary outcomes of the COSMOS study are reported in more detail elsewhere [5,6]. Briefly, the COSMOS study was a 76-week, open-label, multicentre study to evaluate the effects of rosuvastatin on coronary artery atheroma volume, as measured by IVUS, in patients with stable coronary artery disease. Eligible patients were started on 2.5 mg rosuvastatin once daily, which was up-titrated to a maximum of 20 mg/day to achieve a treatment goal of LDL-C <80 mg/dl.

Subjects attended follow-up visits every 4 weeks for 76 weeks after commencing treatment with rosuvastatin. IVUS and coronary angiography (CAG) were performed at baseline and at week 76. All subjects signed an informed consent form. This study was approved by institutional review boards or independent ethics committees at all participating centres.

### Patients

A total of 126 patients completed the COSMOS study and were included in the study database used for this subanalysis. These patients met all of the following inclusion criteria: 20–75 years old; undergoing elective CAG or percutaneous coronary intervention (PCI); serum LDL-C ≥140 mg/dl or TC ≥220 mg/dl in untreated patients, or LDL-C ≥100 mg/dl or TC ≥180 mg/dl in patients already treated with lipid-lowering agents; at least one significant stenosis of ≥75% as a candidate for PCI; and at least one untreated non-culprit target lesion with ≤50% stenosis that could be imaged by IVUS. Exclusion criteria included the following: acute myocardial infarction within 72 h of the start of the study; heart failure of New York Heart Association class III or IV; secondary hyperlipidaemia; treatment with cyclosporine on haemodialysis; left main coronary artery disease with >50% stenosis; uncontrolled hypertension (diastolic blood pressure ≥110 mmHg or systolic blood pressure ≥200 mmHg for all measurements during the screening period); uncontrolled diabetes (HbA1c ≥9.5%); active liver disease or liver dysfunction with ≥2.5× the upper limit of normal (ULN) level for alanine aminotransferase, aspartate aminotransferase or alkaline phosphatase, or total bilirubin ≥3.0 mg/dl; creatinine clearance <30 ml/min or serum creatinine >2.0 mg/dl; and serum creatine kinase >3× the ULN. To ensure the patient population resembled that of actual clinical practice, we did not exclude patients who were taking lipid-lowering drugs at study entry.

### IVUS procedure

IVUS was used to examine plaque volume, lumen volume and vessel volume at baseline and after 76 weeks of treatment. After administering 100–300 μg of intracoronary nitroglycerine, the catheter was advanced into the target vessel and the transducer was positioned as distal to the target lesion as possible. The operator had a motor driving pullback system that progressively withdrew the transducer at a speed of 0.5 mm/second. A Clearview®, Galaxy™ or Galaxy2™ ultrasound system with the Atlantis™ SR Pro 2 40 MHz imaging catheter (Boston Scientific, Natick, MA, USA) was used for both the baseline and follow-up examinations. The images were optimised visually by manipulating the system settings. IVUS images were recorded on super-VHS videotapes or Digital Video Disk plus Re-Writable disks.

### IVUS imaging analysis

Plaque volume was assessed by volumetric analysis using the echoPlaque2 system (Indec Systems Inc., Santa Clara, CA, USA). Baseline and follow-up IVUS images were reviewed side-by-side on a display, and the target segment was selected. The target segment to be monitored was determined in a non-PCI site (>5 mm proximal or distal to the PCI site) with a reproducible feature such as a side branch and its bifurcation, calcifications, or stent edges. A series of cross-sectional images taken every 0.09 mm were measured by manual on-screen planimetry. IVUS tracing was performed in accordance with the standards of the American College of Cardiology and the European Society of Cardiology [16]. Manual planimetry was used to trace the leading edges of the luminal and external elastic membrane (EEM) borders. The images were logged and analysed by two experienced technicians in a central laboratory who were blinded to the patient’s profile, imaging date and baseline/follow-up labels. The accuracy and reproducibility of this method has been established previously [16].

### IVUS measurements

The IVUS parameters analysed in this study were percent change in plaque volume of the target lesion from baseline to follow-up at week 76, percent changes in lumen volume and vessel volume; and changes in plaque area, lumen area and vessel area at the plaque segment with a maximum baseline plaque area.

Percent change in total atheroma volume (TAV) was calculated as follows: percent change in TAV = [TAV(follow-up) – TAV(baseline)]/[TAV(baseline) × 100]. TAV was calculated as the sum of the difference between EEM and luminal area across all evaluated frame images: TAV = ∑(EEM_CSA_ – lumen_CSA_), where CSA = cross-section area. All IVUS measurements were performed at a central laboratory.

### Laboratory tests

All laboratory measurements were performed at a central clinical laboratory (SRL, Inc., Tokyo, Japan). HbA1c (%) is given as National Glycohemoglobin Standardization Program (NGSP) equivalent values (%), which were calculated using the following formula [17]: HbA1c (%) = HbA1c (Japan Diabetes Society value; %) + 0.4%. LDL-C was calculated using Friedewald’s formula [18].

### Statistical analysis

We used the original COSMOS study database for this subanalysis [5]. To identify the factors associated with the percent change in plaque volume, univariate analysis was performed with 70 baseline characteristics and laboratory profiles. Eight factors were significantly associated with the percent change in plaque volume. Multivariate regression analysis was performed using the variables shown to be significant in univariate analyses with stepwise model selection using p < 0.05 to retain variables in the model.

We next divided the subjects into two groups according to HbA1c (low, <6.5%; high, ≥6.5%) based on the American Diabetes Association criteria [19]. Continuous variables were compared between the two groups using two-sample *t*-tests, while comparisons between baseline and follow-up were made using one-sample *t*-tests. Categorical variables were compared between the two groups using *χ*^2^ or Fisher’s exact tests. Finally, the general linear model was used to examine the relationship between change in plaque volume and HbA1c. The two-sided significance level was set at 5%.

Analyses were performed using SAS software, version 9.1.3 (SAS Institute Inc., Cary, NC, USA). Efficacy data are reported as means ± SD.

## Results

As previously reported, 126 patients completed the 76-week study and changes in lipid levels and plaque volume following rosuvastatin treatment were successfully measured [5]. The mean rosuvastatin dose at follow-up IVUS was 16.9 ± 5.3 mg/day, and 92/126 patients (72.2%) were on the maximum rosuvastatin dose (20 mg/day). In these patients (mean ± SD; age 62.6 ± 7.7 years, BMI 25.0 ± 3.3 kg/m^2^, 76.2% males, 37.3% had diabetes), the percent changes in LDL-C and HDL-C were −38.6 ± 16.9% and +19.8 ± 22.9% (both, p < 0.0001). The percent change in plaque volume was −5.1 ± 14.1% (p < 0.0001). HbA1c increased slightly but not significantly from 5.92 ± 0.98% at baseline to 6.25 ± 1.00% at follow-up (p = 0.3205).

### Determinants of plaque progression

The results of univariate analyses to identify factors associated with change in plaque volume are shown in Table 1. BMI, HbA1c and the use of sulfonylureas were positively associated with plaque progression. In contrast, the use of ACE inhibitors, plaque length and plaque volume were negatively associated with plaque progression. The presence of diabetes mellitus was not significantly associated with change in plaque volume. Multivariate analysis (Table 2) revealed that HbA1c and plaque volume at baseline were independent determinants of the change in plaque volume.

**Table 1:** Univariate analyses for the determinants of plaque progression

**Factor**	**β**	**95% CI**	**p Value**
BMI	0.766	0.017, 1.514	0.045
Use of ACE inhibitors	−6.655	−12.604, –0.706	0.0286
Use of sulfonylureas	6.672	0.100, 13.244	0.0467
HbA1c	2.941	0.452, 5.429	0.0209
Evaluated plaque length	−0.997	−1.698, –0.296	0.0057
Plaque volume	−0.112	−0.174, –0.049	0.0006
Vessel volume	−0.047	−0.080, –0.013	0.0067
Plaque area	−0.918	−1.598, –0.238	0.0086

BMI, body mass index.

Parameters with p values <0.05 by univariate analysis are listed.

**Table 2:** Multivariate analysis for the determinants of plaque progression

**Factor**	**β**	**95% CI**	**p Value**
HbA1c	2.683	0.292, 5.074	0.0282
Plaque volume	−0.107	−0.169, –0.046	0.0008

Parameters with p values <0.05 by multivariate analysis on parameters in Table 1 are listed.

### Characteristics of the high and low HbA1c groups

The 126 patients were divided into those with HbA1c <6.5% (n = 80; low) and those with HbA1c ≥6.5% (n = 46; high), with mean HbA1c levels at baseline of 5.35 ± 0.32 and 7.14 ± 0.80%, respectively (p < 0.0001). The baseline characteristics of the two groups were generally similar except for the prevalence of diabetes mellitus, which was higher in the high HbA1c group than in the low HbA1c group, as would be expected. As a result, more patients in the high HbA1c group had received treatment with oral antidiabetic drugs/insulin. Although the location of the lesion was significantly different between the two groups, this factor was not associated with the change in plaque volume. Other characteristics were not significantly different between the two groups (Table 3).

**Table 3:** Baseline characteristics of patients stratified according to HbA1c at baseline

**Characteristic**	**HbA1c <6.5% (n = 86)**	**HbA1c ≥6.5% (n = 40)**	**p Value**
Males	65 (75.58%)	31 (77.50%)	1.0000^a^
Age (years)	62.5 ± 7.9	62.8 ± 7.4	0.8390^b^
Body weight (kg)	64.81 ± 10.63	67.65 ± 14.51	0.2176^b^
BMI (kg/m^2^)	24.68 ± 2.71	25.67 ± 4.23	0.1186^b^
Lesion length	11.095 ± 3.398	10.213 ± 3.548	0.1835^b^
Lesion location			
Proximal	15 (17.44%)	18 (45.00%)	
Distal	33 (38.37%)	7 (17.50%)	
Other	38 (44.19%)	15 (37.50%)	
Target vessel			
RCA	35 (40.70%)	16 (40.00%)	
LAD	25 (29.07%)	13 (32.50%)	
LCX	26 (30.23%)	10 (25.00%)	
LMT	0 (0.00%)	1 (2.50%)	
LLT before enrolment	62 (72.09%)	30 (75.00%)	0.8308^a^
Hypertension	65 (75.58%)	31 (77.50%)	1.0000^a^
Smoking	22 (25.58%)	14 (35.00%)	0.2954^a^
Diabetes mellitus	9 (10.47%)	38 (95.00%)	<0.0001^a^
Family history of CAD	19 (22.09%)	7 (17.50%)	0.6411^a^
Concomitant therapy			
Ca channel blocker	46 (53.49%)	26 (65.00%)	0.2507^a^
Nitrate	57 (66.28%)	24 (60.00%)	0.5512^a^
ACE inhibitor	16 (18.60%)	11 (27.50%)	0.3507^a^
ARB	39 (45.35%)	17 (42.50%)	0.8481^a^
β-blocker	22 (25.58%)	7 (17.50%)	0.3697^a^
Thiazolidinedione	0 (0.00%)	9 (22.50%)	<0.0001^a^
Sulfonylurea	3 (3.49%)	18 (45.00%)	<0.0001^a^
α-glucosidase inhibitor	4 (4.65%)	17 (42.50%)	<0.0001^a^
Insulin	1 (1.16%)	10 (25.00%)	<0.0001^a^
Ticlopidine	83 (96.51%)	38 (95.00%)	0.6522^a^
Clopidogrel	5 (5.81%)	2 (5.00%)	1.0000^a^
Aspirin	86 (100.00%)	40 (100.00%)	1.0000^a^

Data are means ± SD or *n* (%).

^a^*χ*^2^ or Fisher’s exact test; ^b^two-sample t-tests.

RCA, right coronary artery; LAD, left anterior descending; LCX, left circumflex; LMT, left main trunk; LLT, lipid-lowering therapy; CAD, coronary heart disease; HDL-C, HDL-cholesterol; IVUS, intravascular ultrasound; ARB: angiotensin receptor blocker.

### Lipid profiles

LDL-C levels decreased from 140.4 to 81.8 mg/dl (by 39.2%) and from 139.7 to 85.3 mg/dl (by 37.3%) in the high and low HbA1c groups, respectively (Table 4). LDL-C levels at baseline and follow-up and the percent change during the observation period were generally comparable between the two groups. HDL-C levels increased in both groups, although the magnitude of increase was slightly, but significantly, greater in patients with HbA1c <6.5% at baseline. VLDL-C decreased by 4.1% in the low HbA1c group, but increased by 18.1% in the high HbA1c group.

**Table 4:** Changes in laboratory data in patients stratified according to HbA1c at baseline

	**HbA1c <6.5% (n = 86)**				**HbA1c ≥6.5% (n = 40)**			
	**Baseline**	**Follow-up**	**Actual change**	**% change**	**Baseline**	**Follow-up**	**Actual change**	**% change**
TC (mg/dl)			214.6 ± 34.5	157.1 ± 20	−57.6 ± 36.9	−25.3 ± 13.9	211.3 ± 35.4	159.3 ± 31.5	−52 ± 34.9	−23.5 ± 15
TG (mg/dl)			145.6 ± 90.3	121 ± 56.2	−24.6 ± 71.1	−8.9 ± 34.7	152.4 ± 75.9	150.4 ± 76.6*	−2.0 ± 76.2	4.0 ± 44.6
HDL-C (mg/dl)			47.9 ± 10.7	57.0 ± 11.6	9.2 ± 9.0	21.5 ± 22.3	45.4 ± 11.1	51.3 ± 11^†^	6.0 ± 10.2	16.0 ± 24
LDL-C (mg/dl)			140.4 ± 32.3	81.8 ± 15.7	−58.7 ± 33.1	−39.2 ± 16.6	139.7 ± 30.1	85.3 ± 23.9	−54.4 ± 31.2	−37.3 ± 17.7
VLDL-C (mg/dl)			25.4 ± 16.7	19.6 ± 10.4	−5.8 ± 14.2	−4.1 ± 61.8	26.7 ± 16.9	26.5 ± 15^†^	−0.2 ± 16.9	18.1 ± 76.9
non-HDL-C (mg/dl)			166.7 ± 33.9	100 ± 16.9	−66.7 ± 33.5	−38.2 ± 13.9	166 ± 33.1	108 ± 28.5	−58 ± 33	−33.6 ± 17.0
small dense LDL			0.36 ± 0.04	0.35 ± 0.03	−0.01 ± 0.04	−2.77 ± 10.97	0.36 ± 0.04	0.35 ± 0.03	−0.01 ± 0.05	−2.31 ± 11.48
RLP-C (mg/dl)			5.7 ± 5.6	3.6 ± 1.4	−2.1 ± 5.2	−17.3 ± 38.2	5.7 ± 3.7	4.9 ± 2.9^†^	−0.8 ± 3.9	−4 ± 51.3
LDL-C/HDL-C			3.1 ± 1.0	1.5 ± 0.4	−1.6 ± 0.8	−49.1 ± 14.1	3.2 ± 0.9	1.7 ± 0.5^†^	−1.5 ± 0.9	−44.3 ± 16.8
hs-CRP (ng/ml)			2836 ± 7045	821 ± 1331	−2014 ± 7111	−16.2 ± 104.7	4494 ± 9277	1172 ± 1934	−3322 ± 9158	91.9 ± 489.7

Data are means ± SD.

*p < 0.05 and ^†^p < 0.01 vs patients with HbA1c <6.5%.

HDL-C, HDL-cholesterol; LDL-C, LDL-cholesterol; VLDL-C, VLDL-cholesterol; hs-CRP, high-sensitive C-reactive protein.

### IVUS parameters

There were no significant differences in the baseline IVUS parameters (plaque volume, lumen volume and vessel volume) between the two groups of patients (Table 5). Plaque volume decreased from 71.0 ± 39.9 to 64.7 ± 34.7 mm^3^ (by 6.8%) in the low HbA1c group (p < 0.0001 vs baseline) and from 74.3 ± 34.2 to 71.4 ± 32.3 mm^3^ (by 1.3%) in the high HbA1c group. As a result, the percent change in plaque volume was significantly greater in the low HbA1c group than in the high HbA1c group (p = 0.0410). Similarly, vessel volume decreased by 0.8% in the low HbA1c group but increased in the high HbA1c group (by 4.2%), resulting in a significant difference between the two groups in terms of percent change in vessel volume (p = 0.02). In contrast, the change in lumen volume was not significantly different between the two groups (p = 0.08). Changes in percent plaque area and percent vessel area were significantly greater in the low HbA1c group than in the high HbA1c group (both, p < 0.05).

**Table 5:** Changes in IVUS parameters in patients stratified according to HbA1c at baseline

	**HbA1c <6.5% (n = 86)**				**HbA1c ≥6.5% (n = 40)**			
	**Baseline**	**Follow-up**	**Actual change**	**% change**	**Baseline**	**Follow-up**	**Actual change**	**% change**
Volume (mm^3^)								
Plaque	71.0 ± 39.9	64.7 ± 34.7	−6.3 ± 12.3***	−6.8 ± 13.9***	74.3 ± 34.2	71.4 ± 32.3	−2.9 ± 11.4	−1.3 ± 13.8^†^
Lumen	80.3 ± 41.9	82.4 ± 39.8	2.1 ± 11.3	5.6 ± 15.3**	73.9 ± 36.2	80 ± 38.7	6.1 ± 15.6*	10.8 ± 15.8**
Vessel	151.4 ± 75.8	147.1 ± 69	−4.3 ± 18.8*	−0.8 ± 10.7	148.2 ± 65.2	151.4 ± 64.6	3.2 ± 20.5^†^	4.2 ± 13.1*^†^
Area (mm^2^)								
Plaque	8.6 ± 3.7	6.4 ± 3.1	−2.2 ± 2.1***	−24.4 ± 20.5	9.5 ± 3.1	7.9 ± 2.7^†^	−1.7 ± 1.9***	−16.4 ± 17.9^†^
Lumen	6.0 ± 2.8	6.9 ± 3.2	1.0 ± 1.6***	20.2 ± 29.5	6.2 ± 2.4	7.4 ± 3	1.2 ± 1.6***	21.8 ± 26.4
Vessel	14.6 ± 5.7	13.4 ± 5.2	−1.2 ± 2.3***	−7.6 ± 14.4	15.7 ± 4.6	15.3 ± 4.5^†^	−0.5 ± 2.2	−2.0 ± 14.6^†^

Data are means ± SD.

*p < 0.05, **p < 0.01, ***p < 0.0001 vs baseline; ^†^p < 0.05 vs patients with HbA1c <6.5%.

IVUS, intravascular ultrasound.

### Correlation between HbA1c and IVUS parameters

**Figure 1:**
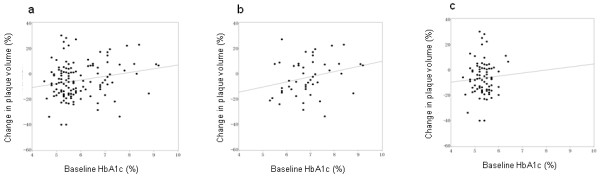
**Associations between HbA1c and plaque volume change.** Associations in all patients (**a**), in patients with diabetes mellitus (**b**), and in non-diabetic patients (**c**).

There was a weak but statistically significant correlation between baseline HbA1c and change in plaque volume (r = 0.206, p = 0.02) (Figure 1a). Because the low HbA1c group contained some patients with diabetes but good glycaemic control, we subdivided both groups of patients according to the presence/absence of diabetes to further examine the role of glycaemic control in diabetic patients. Interestingly, the correlation between baseline HbA1c and change in plaque volume was still apparent in diabetic patients, although this correlation was not statistically significant (r = 0.263, p = 0.07, Figure 1b). The same correlation was not apparent in non-diabetic patients (r = 0.062, p = 0.58, Figure 1c).

## Discussion

In this subanalysis of the COSMOS study, we found that baseline HbA1c was significantly associated with change in plaque volume. Notably, no significant regression was observed in patients with high HbA1c at baseline. In contrast, significant plaque regression was observed in subjects with low HbA1c at baseline, even in those with diabetes, although the decreases in LDL-C levels were similar in both groups. This suggests that glycaemic control, in addition to LDL-C-lowering, is an important determinant of plaque progression or regression. Prior observational studies have revealed a positive association between the presence of diabetes mellitus and the incidence of cardiovascular disease [13,15]. Several recent trials using IVUS have also indicated that diabetes mellitus is a major determinant of plaque progression [11,20]. For example, Hiro et al. investigated the effect of aggressive LDL-C lowering on coronary atherosclerosis in 230 patients with acute coronary syndrome and found that statin-induced regression of coronary plaque volume was weaker in diabetic patients than in non-diabetic patients [11]. Interestingly, they also found that percent change in plaque volume was significantly correlated with LDL-C in patients with diabetes, but not in non-diabetic patients. Nicholls et al. performed a pooled analysis of five IVUS trials involving 2,237 subjects and compared arterial remodelling, extent of coronary atherosclerosis and disease progression between patients with diabetes and those without [20]. They found that diabetic patients exhibited greater percent and total atheroma volumes, with more rapid progression of plaque volume and inadequate compensatory remodelling. They also found that percent atheroma volume was more strongly associated with HbA1c than with fasting glucose, although this difference became non-significant after adjustment for patient background. Similarly, Berry et al. observed significant associations between fasting glucose, HbA1c and the presence of diabetes mellitus, and the severity or progression of coronary atherosclerosis in 426 patients who underwent IVUS [21]. Similar to these earlier studies, we observed a significant association between baseline HbA1c and change in plaque volume.

The reduced plaque regression in patients with high HbA1c suggests that hyperglycaemia or diabetes mellitus may be involved in a unique pathogenic mechanism underlying plaque formation in these patients. Indeed, hyperglycaemia could accelerate the development of atherosclerosis through enhanced production of advanced glycation end products, oxidative stress and vascular inflammation, which may contribute to diabetes-specific atherosclerosis [22,23]. This is supported by the results of histological studies and imaging studies, which have revealed that several features are more pronounced in diabetic patients, including more extensive macrophage infiltration, significantly larger lipid cores, and more abundant dense-calcium or fibrocalcific tissue [24,25]. More recently, Parathath et al. demonstrated that diabetes modified plaque macrophage characteristics and thus hindered plaque regression [26].

As the relationship between the change in plaque volume and HbA1c was mainly observed in diabetic patients, and not in non-diabetic patients, HbA1c seems to be an important determinant of plaque regression in diabetic patients. Thus, targeting glycaemic control may aid plaque regression. Indeed several clinical trials have demonstrated significant plaque regression using oral antidiabetic drugs. For example, the Pioglitazone Effect on Regression of Intravascular Sonographic Coronary Obstruction Prospective Evaluation (PERISCOPE) trial compared the effects of treatment with pioglitazone or glimepiride for 18 months on plaque volume in 543 patients with coronary disease and type 2 diabetes [8]. The investigators found that the least squares mean percent atheroma volume decreased from baseline in patients treated with pioglitazone but increased in patients treated with glimepiride (−0.16 vs +0.73%, respectively, p = 0.002). HbA1c levels were 7.4 ± 1.0% in both groups at baseline, with greater decreases in patients treated with pioglitazone compared with those treated with glimepiride (−0.55 vs −0.36%, p = 0.03). Pioglitazone, but not glimepiride, was also associated with improvements in lipid levels, including HDL-C and triglycerides, which likely contributed to plaque regression in this group. These data suggest that interventions that improve glycaemic control alone may be insufficient to prevent increases in atheroma volume. Instead, improvements in multiple factors may be necessary to achieve clinically meaningful plaque regression. Clearly, further data from this and other studies are needed to examine the relative contributions of improved control of glucose and lipid levels to plaque regression.

HbA1c, which is an established diagnostic marker for diabetes mellitus [19], has been reported to be a significant predictor of cardiovascular events in diabetic patients [14,15]. In fact, a recent meta-analysis demonstrated that intensive glucose control could reduce the occurrence of cardiovascular events, although longer observation periods than originally expected may be required to examine this association [27].

Quevedo et al. reported a high prevalence of vessel shrinkage in diabetic patients, which was associated with insulin requirement, HbA1c, apolipoprotein B and hypertension in their study using serial IVUS [28]. Nicholls et al. reported inadequate compensatory remodelling in diabetic patients [20]. However, unlike these earlier observations, in the present study, the percent change in vessel volume during the follow-up period indicated positive remodelling in patients with high HbA1c and negative remodelling in patients with low HbA1c. Reddy et al. also observed greater positive remodelling in diabetic patients than in non-diabetic patients [29]. Meanwhile, Chhatriwalla et al. [30] found that lower levels of LDL-C and systolic blood pressure were associated with negative remodelling of the elastic membrane in patients with established coronary artery disease. Differences in patient characteristics and IVUS methodologies may partly explain these differences in vascular remodelling between these studies. Multifactorial treatment strategies targeting not just LDL-C, but also other risk factors, including glycaemic control and blood pressure, may be important to achieve negative remodelling, and slow the progression of coronary atherosclerosis.

We observed a slight increase in HbA1c from 5.92 to 6.25% during the study, although this change was not significant. Some studies have reported that rosuvastatin and other statins increase markers of insulin resistance, such as homeostasis model assessment of insulin resistance (HOMA-IR) and fasting insulin levels [31-33]. It is possible that rosuvastatin reduces insulin sensitivity, resulting in a slight deterioration in glycaemic control. However, this effect of rosuvastatin is not consistent among studies [34-36]. Therefore, we think a more likely explanation is that the change in HbA1c reflects the natural progression of insulin resistance or worsening glycaemic control in a cohort of patients, in which 37.3% had diabetes at baseline. It must also be noted that the earlier studies were much shorter (total study length of 1–3 months) than our study, possibly too short to reliably attribute the changes in HbA1c to rosuvastatin itself. Unfortunately, as we did not measure fasting glucose or fasting insulin, we were unable to determine HOMA-IR as a direct marker for insulin resistance. We should also consider that, although HbA1c is strongly associated with fasting and post-prandial plasma glucose, the use of HbA1c rather than specific glucose parameters may mask possible associations between plasma glucose and both fasting/post-prandial glucose excursions and plaque progression.

It is also important to consider that other factors not assessed here may partly explain some of the observed associations. For example, in a cohort of Korean individuals with normal glucose tolerance or type 2 diabetes, serum levels of the adipokine omentin-1 were independently associated with arterial stiffness and carotid plaque, even after adjusting for other cardiovascular risk factors [37].

In the present study, there were some marked differences in the changes in lipid levels between the two groups. For example, VLDL-C decreased by 4.1% in the low HbA1c group, but increased by 18.1% in the high HbA1c group. The reason for this difference and its clinical relevance are unclear, because VLDL-C was not associated with plaque progression in univariate analyses, and was not included in the multivariate model. Further studies may be required to understand these results and their possible implications.

This subanalysis of the COSMOS study was a post-marketing study designed to investigate the effects of intensive LDL-C lowering with rosuvastatin on coronary atherosclerosis measured using IVUS and its safety; as such, the lack of glucose measurements and the small sample size limit further interpretations of the current results. It is also possible that other factors not assessed here, including insulin and adipokine concentrations, insulin resistance, and other markers of glycaemic control may partly explain the associations observed. Nevertheless, the association between HbA1c and change in plaque volume demonstrates the importance of good glucose control and the unique atherogenic processes in diabetes. The results also support a prospective interventional trial to better understand the impact of optimal glycaemic control on plaque regression.

## Conclusions

This subanalysis of the COSMOS study revealed a significant association between baseline HbA1c and change in plaque volume. In patients with high HbA1c, no significant plaque regression was observed, whereas marked plaque regression was observed in patients with low HbA1c, even in those with diabetes, although the decreases in LDL-C levels were similar in both groups. These findings, together with earlier results, suggest that glycaemic control, along with LDL-C, is an important determinant of plaque progression and regression in patients with stable coronary artery disease.

## Appendix

### Investigators

N. Kawashima, Department of Cardiovascular Medicine, Hokkaido University Graduate School of Medicine, Sapporo; S. Tanaka, Department of Cardiology, Hokkaido Cardiovascular Hospital, Sapporo; O. Hirono, Department of Internal Medicine, Yamagata Prefectural Shinjo Hospital, Shinjo; T. Kubota, Department of Cardiology, Saitama Cardiovascular and Respiratory Center, Kumagaya; T. Ohba, Department of Internal Medicine, Chiba Hokusoh Hospital, Inba; T. Takayama, Division of Cardiovascular Medicine, Nihon University School of Medicine, Tokyo; K. Miyauchi, Department of Cardiology, Juntendo University School of Medicine, Tokyo; S. Tani, Department of Cardiology, Nihon University Surugadai Hospital, Tokyo; S. Ishiwata, Department of Cardiology, Cardiovascular Center, Toranomon Hospital, Tokyo; K. Fukui, Department of Cardiovascular Medicine, Kanagawa Cardiovascular and Respiratory Center, Yokohama; Y. Murakami, Department of Cardiovascular Medicine, Aichi Prefectural Cardiovascular and Respiratory Center, Ichinomiya; K. Nishigaki, Department of Internal Medicine, Gifu University Hospital, Gifu; T. Noda, Department of Cardiology, Gifu Prefectural General Medical Center, Gifu; K. Ueno, Department of Cardiology, Gifu Municipal Hospital, Gifu; T. Kimura, Department of Cardiovascular Medicine, Kyoto University Hospital, Kyoto; H. Nonogi, Division of Cardiology, National Cardiovascular Center, Suita; N. Awata, Department of Cardiology, Osaka Medical Center for Cancer and Cardiovascular Diseases, Osaka; T. Kataoka, Department of Internal Medicine and Cardiology, Osaka City University Medical School, Osaka; A. Itoh, Department of Cardiology, Osaka City Central Hospital, Osaka; Y. Nagai, Department of Cardiology, Rinku General Medical Center, Izumisano; Y. Fukuyama, Department of Cardiovascular Medicine, Kobe City Medical Center General Hospital, Kobe; T. Kawagoe, Department of Cardiovascular Medicine, Hiroshima City Hospital, Hiroshima; T. Hiro, Division of Cardiology Medicine, Department of Medicine and Clinical Science, Yamaguchi University Graduate School of Medicine, Ube; T. Wakiyama, Division of Cardiology, Tokuyama Central Hospital, Shunan; T. Kawasaki, Department of Cardiovascular Medicine, Shinkoga Hospital, Kurume; T. Honda, Cardiovascular Center, Saiseikai Kumamoto Hospital, Kumamoto; K Urasawa, Cardiovascular Center, Tokeidai Memorial Hospital, Sapporo; H. Iida, Department of Cardiology, National Hospital Organization Hamada Medical Center, Hamada; S. Ono, Department of Cardiology, Saiseikai Yamaguchi General Hospital, Yamaguchi; T. Oda, Department of Cardiology, Shimane Prefectural Central Hospital, Izumo; S. Minagoe, Department of Cardiology, National Hospital Organization Kagoshima Medical Center, Kagoshima; K. Hibi, Cardiovascular Center, Yokohama City University Medical Center, Yokohama; S. Yanagi, Department of Cardiology, Seichokai Fuchu Hospital, Izumi; N. Kubo, Cardiovascular Division, Saitama Medical Center, Jichi Medical University, Saitama; M. Kawashiri, Division of Cardiovascular Medicine, Kanazawa University Graduate School of Medical Science, Kanazawa; H. Shimomura, Division of Cardiology, Fukuoka Tokushukai Medical Center, Kasuga; J. Yajima, Department of Cardiology, The Cardiovascular Institute, Tokyo, Japan. (Affiliations written here are correct as of the end of the primary study).

## Abbreviations

ARB, Angiotensin receptor blocker; CAD, Coronary artery disease; CAG, Coronary angiography; COSMOS, Coronary Atherosclerosis Study Measuring Effects of Rosuvastatin Using Intravascular Ultrasound in Japanese Subjects; CSA, Cross-section area; EEM, External elastic membrane; hs-CRP, High-sensitive C-reactive protein; IVUS, Intravascular ultrasound; LAD, Left anterior descending artery; LCX, Left circumflex artery; LLT, Lipid-lowering therapy; LMT, Left main coronary artery; NGSP, National Glycohemoglobin Standardization Program; PCI, Percutaneous coronary intervention; RCA, Right coronary artery; TAV, Total atheroma volume; ULN, Upper limit of normal.

## Competing interests

H. Daida has received consulting fees and support for travel to meetings from AstraZeneca K.K. and Shionogi & Co., Ltd; acted as a consultant for GlaxoSmithKline K.K., Kowa Pharmaceutical Co., Ltd., Sanofi-Aventis K.K. and Schering-Plough Corp; received grants from Astellas Pharma Inc., AstraZeneca K.K., Bayer Yakuhun Ltd., Dainippon Sumitomo Pharma Co., Ltd., Kissei Pharmaceutical Co., Ltd., Kowa Pharmaceutical Co., Ltd., Kyowa Medex Co., Ltd., MSD K.K., Novartis Pharma K.K., Nippon Boehringer Ingelheim Co., Ltd., Otsuka Pharmaceutical Co., Ltd., Public Health Research Foundation, Roche Diagnostics K.K., Sanofi-Aventis K.K., Sanwa Kagaku Kenkyusho Co., Ltd., Schering-Plough Corp., Shionogi & Co., Ltd., Takeda Pharmaceutical Co., Ltd., and The Waksman Foundation of Japan, Inc.; and served on the speakers bureaus of Astellas Pharma Inc., Bayer Yakuhin Ltd., Dainippon Sumitomo Pharma Co., Ltd., Kissei Pharmaceutical Co., Ltd., Kowa Pharmaceutical Co., Ltd., MSD K.K., Novartis Pharma K.K., Nippon Boehringer Ingleheim Co., Ltd., Sanofi-Aventis K.K., Sanwa Kagaku Kenkyusho Co., Ltd., Schering-Plough Corp. and Takeda Pharmaceutical Co., Ltd. The other authors report no conflicts of interest.

## Author contributions

HD drafted the manuscript; TT, TH and MY conceived of and designed the study; AY and SS contributed to the analysis of the data; TY collected data; and MM coordinated and managed the study. All authors read and approved the final manuscript.
